# The seasonal dynamics and biting behavior of potential *Anopheles* vectors of *Plasmodium knowlesi* in Palawan, Philippines

**DOI:** 10.1186/s13071-021-04853-9

**Published:** 2021-07-07

**Authors:** Richard Paul B. Malijan, Frank Mechan, Jessie C. Braganza, Kristelle Mae R. Valle, Ferdinand V. Salazar, Majhalia M. Torno, Wilfredo E. Aure, Brian A. Bacay, Fe Esperanza Espino, Stephen J. Torr, Kimberly M. Fornace, Chris Drakeley, Heather M. Ferguson

**Affiliations:** 1 Department of Medical Entomology, Research Institute for Tropical Medicine, Alabang, 1781 Muntinlupa City, Metro Manila Philippines; 2 Department of Vector Biology, Liverpool School of Tropical Medicine, Liverpool, L3 5Q4 UK; 3 Present Address: Taxonomy & Pesticide Efficacy Branch, Vector Biology & Control Division, Environment Health Institute, National Environment Agency, Ministry of Sustainability and the Environment, 11 Biopolis Way, Singapore, 138667 Singapore; 4 Department of Parasitology, Research Institute for Tropical Medicine, Alabang, 1781 Muntinlupa City, Ma, Metro Manila Philippines; 5 Faculty of Infectious and Tropical Diseases, London School of Tropical Medicine and Hygiene, London, WC1E 7HT UK; 6 Institute of Biodiversity, Animal Health and Comparative Medicine, University of Glasgow, Glasgow, G12 8QQ UK

**Keywords:** *Anopheles balabacensis*, *Anopheles flavirostris*, *Plasmodium knowlesi*, Vector behavior, Philippines

## Abstract

**Background:**

A small number of human cases of the zoonotic malaria *Plasmodium knowlesi* have been reported in Palawan Island, the Philippines. Identification of potential vector species and their bionomics is crucial for understanding human exposure risk in this setting. Here, we combined longitudinal surveillance with a trap-evaluation study to address knowledge gaps about the ecology and potential for zoonotic spillover of this macaque malaria in Palawan Island.

**Methods:**

The abundance, diversity and biting behavior of human-biting *Anopheles* mosquitoes were assessed through monthly outdoor human landing catches (HLC) in three ecotypes representing different land use (forest edge, forest and agricultural area) across 8 months. Additionally, the host preference and biting activity of potential *Anopheles* vectors were assessed through comparison of their abundance and capture time in traps baited with humans (HLC, human-baited electrocuting net—HEN) or macaques (monkey-baited trap—MBT, monkey-baited electrocuting net—MEN). All female *Anopheles* mosquitoes were tested for the presence of *Plasmodium* parasites by PCR.

**Results:**

Previously incriminated vectors *Anopheles balabacensis* and *An. flavirostris* accounted for > 95% of anophelines caught in longitudinal surveillance. However, human biting densities were relatively low (*An. balabacensis*: 0.34–1.20 per night, *An. flavirostris*: 0–2 bites per night). Biting densities of *An. balabacensis* were highest in the forest edge, while *An. flavirostris* was most abundant in the agricultural area*.* The abundance of *An. balabacensis* and *An. flavirostris* was significantly higher in HLC than in MBT. None of the 357 female *Anopheles* mosquitoes tested for *Plasmodium* infection were positive.

**Conclusions:**

The relatively low density and lack of malaria infection in *Anopheles* mosquitoes sampled here indicates that exposure to *P. knowlesi* in this setting is considerably lower than in neighboring countries (i.e. Malaysia), where it is now the primary cause of malaria in humans. Although anophelines had lower abundance in MBTs than in HLCs, *An. balabacensis* and *An. flavirostris* were caught by both methods, suggesting they could act as bridge vectors between humans and macaques. These species bite primarily outdoors during the early evening, confirming that insecticide-treated nets are unlikely to provide protection against *P. knowlesi* vectors.

**Graphical abstract:**

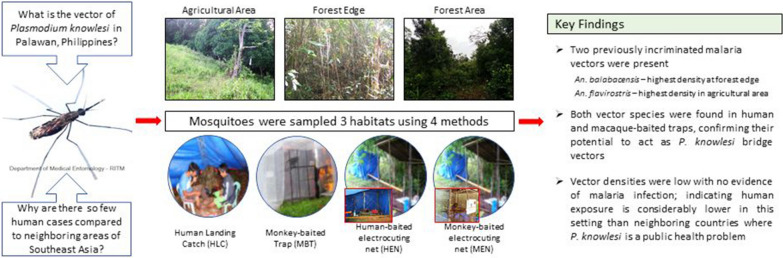

**Supplementary Information:**

The online version contains supplementary material available at 10.1186/s13071-021-04853-9.

## Background

The Philippines has established a subnational/territorial malaria elimination strategy, through which zero indigenous cases were reported in 78 out of 81 provinces in 2019 [1–3]. The primary malaria species of public health importance in the Philippines are *Plasmodium falciparum* and *P. vivax* which respectively comprise ~ 88% and 9% of the total indigenous malaria cases [1]. Malaria transmission in the country is now confined to a few provinces including Palawan [1–3]. Concern has been raised that the emergence of the zoonotic malaria parasite *P. knowlesi* as a public health problem in several Southeast Asian countries may threaten regional elimination [4, 5]. Human cases of *P. knowlesi* infection in Palawan, Philippines were first confirmed in 2008, based on molecular detection from blood slides that had been previously diagnosed by microscopy as *P. malariae* [6]. Recent serological work indicates that *P. knowlesi* transmission in Palawan is ongoing, with community sampling reporting that 1.1% of individuals tested positive for the *P. knowlesi*-specific PkSERA3 ag1 antibody response [7]. In response to the emerging threat of *P. knowlesi*, an international collaboration was established in 2012 to investigate the risk factors for human infections and identify populations at risk. The MONKEYBAR project focused investigation on two known areas of transmission: Sabah in Malaysian Borneo and Palawan Island in the Philippines [7, 8]. Although human infections of *P. knowlesi* have been reported in both settings [5, 7, 9], cases have been sporadic in Palawan [6, 10, 11] whilst *P. knowlesi* is now the leading cause of human malaria in Sabah [5, 12, 13].

The primary reservoirs of *P. knowlesi* are the long-tailed (*Macaca fascicularis*) and pig-tailed (*M. nemestrina*) macaques that are widely distributed throughout Southeast Asia [14, 15]. Long-tailed macaques are the only monkey species in the Philippines, and are widely distributed throughout the country including Palawan [16]. While long-tailed macaques have been confirmed as reservoirs of *P. knowlesi* in Palawan [17], there is limited understanding of the ecology of *P. knowlesi* transmission and potential for human spillover in this setting. Of particular concern is whether human *P. knowlesi* cases will continue to be sporadic and rare in Palawan, or will transition into substantial spillover into human populations as has occurred in the neighboring area of Sabah, Malaysian Borneo; which is < 100 km across the sea from Palawan. Variation in epidemiological potential may be related to differences in vector species and their interactions with human and macaque host species. Understanding the local ecology of transmission is vital to identify both spillover potential and control strategies [14].

Competent *Anopheles* species that feed on both human and monkey hosts could act as *P. knowlesi* bridge vectors [15, 18]. Mosquitoes in the *Anopheles leucosphyrus* group have been implicated as *P. knowlesi* vectors and capable of cross-species transfer between macaques to humans [12, 18–21]. Primary vector species vary geographically [22–25], with *An. balabacensis* and *An. donaldi* being the most important in Sabah [26, 27]. In the Philippines, there has been relatively limited investigation of *Anopheles* vectors of simian malaria. Early work (1970s) indicated that *An. balabacensis* is the most likely vector of simian malaria on Palawan [28]; however, there has been no recent confirmation of the role of this vector within the period of *P. knowlesi* emergence in humans.

Investigating the ecology and behavior of potential vectors of *P. knowlesi*, and incrimination of the vector species responsible for cross-species transmission are essential to identify human populations at risk and develop appropriate control strategies [29]. The gold standard method for directly measuring human exposure to malaria vectors is the human landing catch (HLC) [30, 31]. However, this approach raises ethical concerns by exposing people to mosquitoes that might be infected with mosquito-borne diseases; many of which have no or limited prophylactic and treatment options. Previously, monkey-baited traps (MBT) have been used as the reference method for estimating mosquito biting rates on monkeys; however estimates from this approach are not directly comparable to HLC due to differences in procedures, and it raises animal welfare concerns [15, 19, 25]. The development of alternative mosquito trapping methods that can provide more standardized comparisons of mosquito attraction to humans and macaques, without risking host exposure to infection, would be of great value.

Electrocuting traps may offer a solution to some issues associated with traditional mosquito trapping methods [36] by using host odor to attract and sample mosquitoes [37, 38]. Such traps were originally used to sample tsetse flies in Africa and similar traps using host odor have been evaluated for mosquitoes [32, 33]. One type of electrocuting trap, the electric net (E-net), was recently evaluated for sampling mosquito vectors of *P. knowlesi* in Sabah, Malaysia [19]. Here, E-nets baited with humans and macaques generally had poorer performance than HLCs, but higher than MBTs. The potential for wider application of E-nets as a general surveillance tool for zoonotic malaria vectors has yet to be demonstrated.

Here we combined longitudinal surveillance of potential *P. knowlesi* vectors in Palawan Island with a trap-evaluation study to identify potential vector species and investigate how their ecology and infection varied between ecotypes. The aims of the longitudinal study were to characterize the abundance, diversity, seasonal dynamics, biting behavior and *Plasmodium* spp. infection rates of potential human-biting vectors in three different habitats: forest, forest edge and agricultural. This study also aimed to assess different trapping methods for sampling human- and macaque-biting vectors in order to identify species that could act as bridge vectors.

## Methods

### Study site

**Fig. 1:**
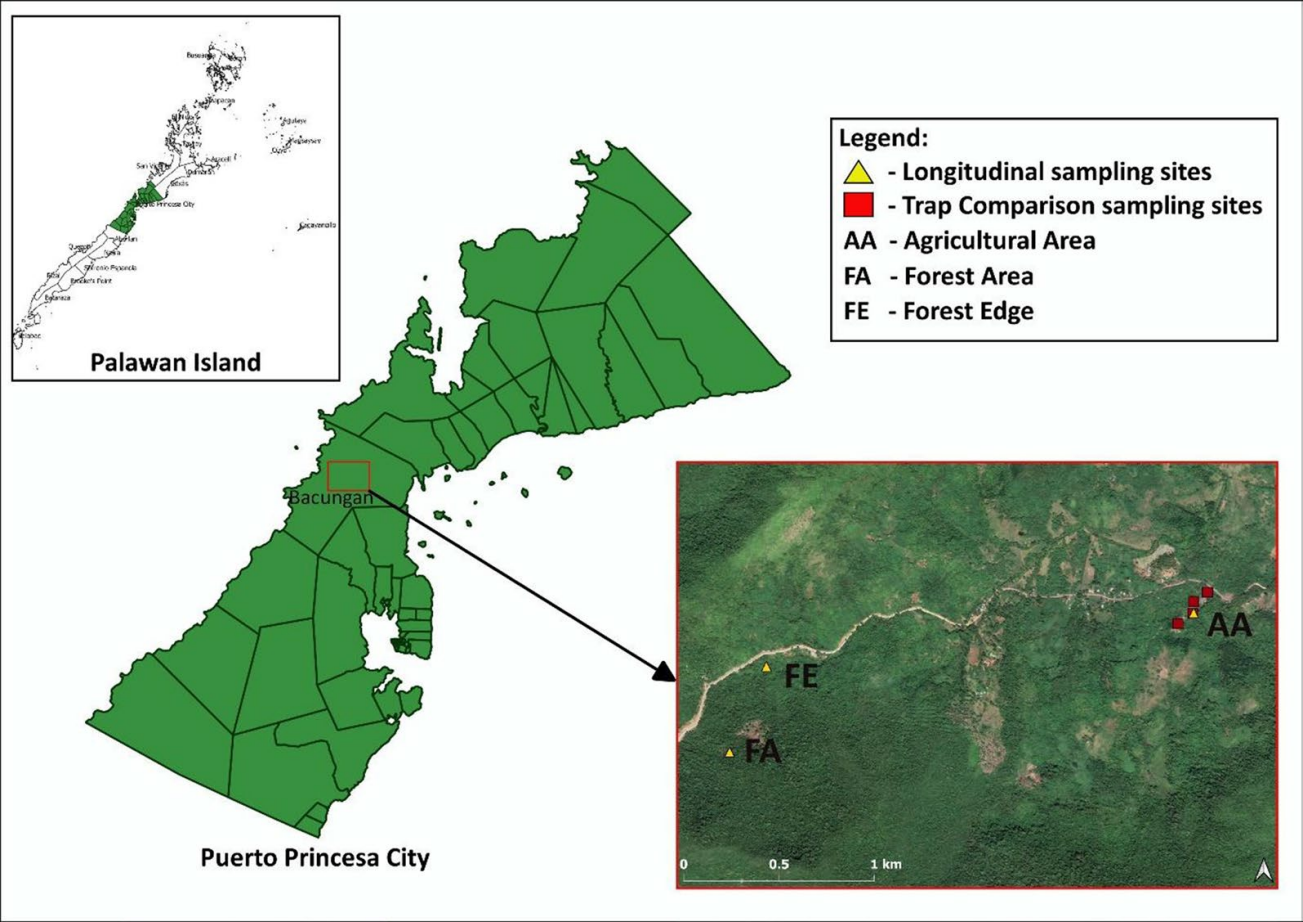
Map showing the location in Brgy. Bacungan, Puerto Princesa City, Palawan, and the sampling sites labeled AA, FA and FE

Two separate field experiments were conducted in Barangay (Brgy.) Bacungan, Puerto Princesa City, Palawan in 2015: (1) a longitudinal study of human-biting mosquitoes and (2) a comparison of human- and monkey-baited traps (Fig. 1). Barangay Bacungan is an area with intact secondary forest and some remaining primary forest. This study site was selected based on the locations of previously reported human *P. knowlesi* cases and was the site of integrated entomology, primatology and social studies within a wider research program on risk factors for *P. knowlesi* [6, 7, 9].

The presence of *An. balabacensis*, a vector of *P. knowlesi* [27, 28, 34], was confirmed from pilot mosquito collections as well as sightings of long-tailed macaques (*M. fascicularis*)*.* The relative accessibility and safety of the area year-round were also considered in the selection of the sites. Experiments were conducted between May and December 2015, coinciding with the northeast monsoon season of high rainfall.

### Trapping techniques

Four trapping techniques were used in this study to characterize the behavior of potential *Anopheles* vectors in Palawan.

### Human landing catch (HLC)

**Fig. 2:**
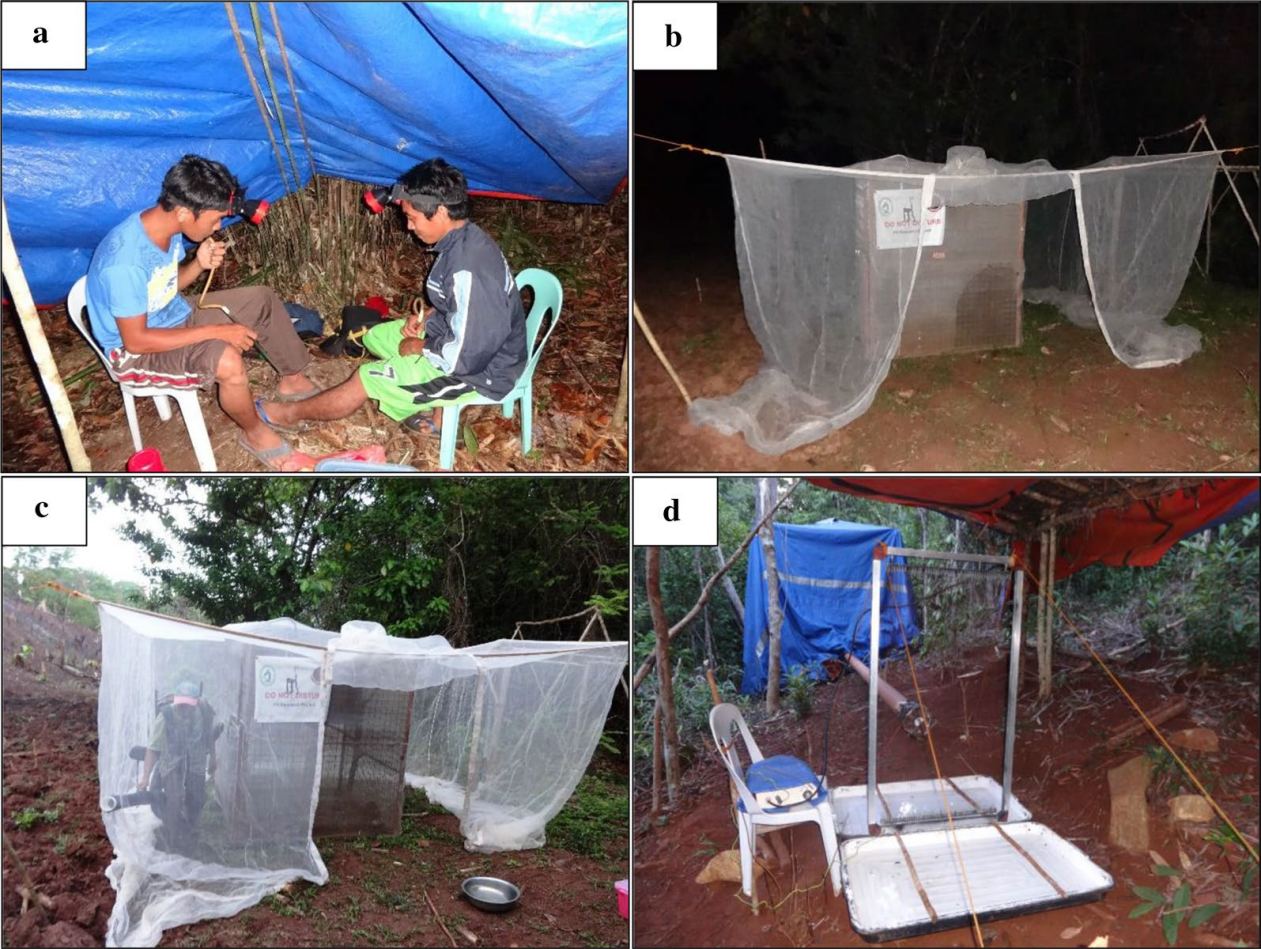
Mosquito collection techniques used in the study: **a** human landing catch (HLC), **b** monkey-baited trap (MBT), **c** human collectors obtaining mosquitoes from the MBT, **d** electrocuting net trap (HEN and MEN)

Human landing catches were performed outdoors from 18:00 to 06:00. All trained HLC collectors were male residents in the study site, aged between 20 and 40 years. For the longitudinal study, two collectors performed the HLC in a pair, wherein one individual exposed their bare legs while seated (Fig. 2a) and the second used a manual aspirator to collect any mosquitoes that landed on the other’s legs. Aspirated mosquitoes were transferred into separate collection cups labeled with the sampling station and hour of collection. At midnight, the collectors swapped roles so that each individual performed as both collector and bait over the course of the night. This protocol was modified slightly for the trap evaluation study, where only one person carried out each HLC, acting as both bait and collector.

### Monkey-baited trap (MBT)

In previous studies, monkey-baited traps have been used as the reference method to sample mosquitoes attracted to macaques [15, 19, 25]. In this study, one adult female long-tailed macaque (*M. fascicularis*) was placed inside a steel cage measuring 2.0 × 2.0 × 2.0 m and fitted with wire mesh to prevent entry of mosquitoes. The origin of macaques used in this study and their holding conditions are described in Additional file 1.

During mosquito trapping, a large untreated mosquito net (3.9 m × 3.0 m × 3.3 m) was suspended around the cage with the door flap open (Fig. 2b). Mosquitoes attracted to the macaque entered the outer net but the wire mesh of the monkey cage prevented them from feeding on the macaque. This internal protective net was not used in most previous work with MBTs [15, 25, 34], but was incorporated into MBT design here and in another recent study by Hawkes et al. [19] in Sabah, Malaysia, as a requirement of the ethics approval granted to work with primates. Mosquitoes resting between the cage and outer net were collected every hour from 18:00 to 06:00 using a CDC backpack aspirator (Fig. 2c).

### Electrocuting nets (HEN and MEN)

Electrocuting net traps work by piping the scent from a single host, housed in an enclosed tarpaulin tent, out to a collection point that is covered with an electrified surface. Two versions of this trap were used in this study: a human-baited electrocuting net (HEN) and a monkey-baited electrocuting net (MEN). In the HEN, the tent contained a human male (same volunteers as in the HLC collections) while in the MEN the tent contained a female long-tailed macaque (same macaques as participated in MBT). The host’s scent was pumped from the tent to an electrified grid (Fig. 2d) via a 6-m PVC pipe using a co-axial fan (120 × 120 × 25 mm, 3100 RPM speed, air volume 3.229 m^3^/min). The electrified grid, measuring 1 m tall by 0.5 m wide, consisted of vertically-arranged copper wires (0.2 mm thick) spaced 5 mm apart. Alternate wires in each bank were charged by a transformer with a DC input of 12 V (3 amps) and an output of ~ 50 kV pulsing at ~ 70 Hz. Mosquitoes stunned by the electrified grid were collected in pans with water and liquid soap (to break the surface tension of the water and allow the mosquitoes to sink before escape), each pan extending 44 cm from each side of the electrified grid. The electrocuting net trap was used to collect mosquitoes from 18:00 to 06:00, with mosquito specimens collected once from the pan at the end of the night and transferred into a collection cup containing 70% ethanol. The electrified grids were also inspected for any mosquitoes attached to the wires.

### Longitudinal study of human-biting mosquitoes

This study investigated the abundance and biting activity of *Anopheles* mosquitoes at three sites on Palawan Island between May and December 2015. Each collection site was broadly representative of an ecotype in Brgy. Bacungan: agricultural area (9° 53.320′ N, 118° 39.076′ E), forest edge (9° 53.167′ N, 118° 37.850′ E) and forest area (9° 52.921′ N, 118° 37.744′ E). There was a minimum distance between sites of 1 km (Fig. 1). A GPS device (Garmin 62SC) was used to ensure that collections were conducted repeatedly in the same spot.

The agricultural area was cleared land used for small-scale, low-input farming of mixed crops of fruit-bearing trees (mango, cashew, jackfruit) and upland rice. The forest edge area was located at the margin between secondary forest and a cleared agricultural area, characterized by a mixture of small trees and shrubs and surrounded by bamboo clusters. The forest area (secondary forest) was characterized by having more than 10% canopy cover, presence of tree species with a minimum height of 5 m, and no or low anthropogenic disturbances.

Monthly mosquito collections were carried out simultaneously at the three sites for three consecutive nights between May and December 2015. On each night, HLC collections were conducted hourly between 18:00 and 06:00. Simultaneous collections were made at each site on each night of collection by three separate teams. These teams rotated between sampling sites each night.

### Comparison of human- and monkey-baited traps

This study was designed to compare mosquito collection techniques that use human and macaque hosts. The aim was to characterize the host preference of *Anopheles* species including potential *P. knowlesi* vectors by contrasting their relative abundance in traps baited with humans versus macaques. Outdoor collections of human- and monkey-biting mosquitoes were conducted using HLC, MBT, HEN and MEN at four collection stations: I (118° 39.076′ E, 9° 53.353′ N), II (118° 39.116′ E, 9° 53.368′ N), III (118° 39.074′ E, 9° 53.320′ N) and IV (118° 39.031′ E, 9° 53.290′ N).

To minimize the influence of environmental factors, all collection sites were located within the same agricultural area, with each sampling station spaced approximately 100 m apart in a Latin square design. Collections using each of the four trapping methods were conducted simultaneously from 18:00 to 06:00, with one trap at each of the four collection stations. Traps were rotated between stations each night to give a complete replicate every four collection nights. These 4-day replicates were carried out over 40 non-continuous nights between May to July 2015, providing a total of 10 full replicates of each trap in each collection station.

### Mosquito processing and identification

Mosquitoes captured within the same one-hour period were stored together in a holding cup and labeled by hour, collection site and trap type used. A field supervisor visited the teams hourly to gather and replace the collection cup. Immediately upon collection, mosquitoes were killed using ethyl acetate then placed in a cell culture plate (12-well; 12.5 × 8.5 × 2 cm) which was subdivided by time of collection.

All collected mosquitoes were taken to a field laboratory the day after the collection night for morphological identification. All mosquitoes (male and female) were identified to genus level based on morphology. Female *Anopheles* mosquitoes were identified further to species level using illustrated keys [40], while non-anopheline mosquitoes (male and female) were segregated by genus level and later identified to species level in the field laboratory [35]. After identification, all mosquito samples were placed in 1.5 ml microcentrifuge tubes lined with filter paper and silica gel. For samples collected using E-nets, mosquitoes were placed in 1.5 ml microcentrifuge tubes with 70% ethanol instead of filter paper and silica gel as stunned mosquitoes had already been soaked in a water pan.

Each microcentrifuge tube was labeled with a unique collection number corresponding to the date of collection, the time of collection, the collection station, trap type and initial species identification. Validation of *Anopheles* morphological identification was conducted by entomologists at the Research Institute for Tropical Medicine (RITM), Muntinlupa City, Metro Manila. All samples were stored in an incubator (Thermo Fisher Scientific) at 37 °C prior to processing for molecular analysis.

### Molecular detection of *Plasmodium* in mosquitoes

All female *Anopheles* mosquitoes collected during the study were screened for malaria parasites. The head and thorax of dried female *Anopheles* specimens were separated from the rest of their body and placed individually in separate microcentrifuge tubes. For HEN and MEN collections, the ethanol used for mosquito preservation was allowed to evaporate completely by placing sample tubes in an AccuBlock dry bath (Labnet, USA) set at 70 °C, with whole mosquito specimens used for DNA extraction. Genomic DNA was extracted from the head and thorax of each mosquito using the DNeasy tissue kit (Qiagen, Germany) according to the manufacturer’s protocol. Eluted DNA from the same mosquito species collected from the same trap was pooled in a separate microcentrifuge tube (maximum of 10 eluted DNA per pool) and kept in a freezer at −20 °C until required.

Detection of malaria parasites from the pooled specimens was conducted using a nested PCR assay using primers based on the *Plasmodium* small subunit ribosomal RNA (SSU rRNA). Primers and protocols used for *Plasmodium* detection were as described by Singh et al. [36]. For *Plasmodium*-positive pools, the first nested PCR assay was performed again for each sample from the pool. A second nested PCR assay was performed on the *Plasmodium*-positive samples to determine the species using nine species-specific primers (Additional file 2).

Nested PCR assays were performed with 25 µl final volume consisting of 5.0 µl of 5X PCR buffer (Promega), 0.5 µl of dNTP (10 mM) mixture (Promega), 3.0 µl of 25 mM MgCl_2_ (Promega), 1.0 µl each of 10 µM forward and reverse primers, 0.3 µl of Taq DNA polymerase (5 U/µl), 2.0 µl of the DNA template and sterile dH_2_O up to 25 µl final volume.

The PCR conditions used were as follows: an initial denaturation at 95 °C for 5 min, followed by 35 cycles of 94 °C for 1 min, annealing for 1 min and 72 °C for 1 min, and a final extension at 72 °C for 5 min. The annealing temperature was set based on the optimum temperature of the primers (Additional file 1). After completion of the first PCR, 2.0 µl of the first PCR product was used as a template in the second PCR.

### Statistical analysis

Statistical analysis was conducted using the R programming language (version 3.2.3). Generalized linear mixed models (GLMM) were constructed to analyze the variables of interest (nightly or hourly mosquito abundance) using key explanatory variables of collection site, hour and month (for longitudinal study) or trapping method and host bait (for trap comparison study). Graphs were produced using ggplot2 (version 2.2.1). All confidence intervals were estimated with bootstrap resampling of 10,000 samples using the ‘boot’ package (version1.3-19).

Stepwise regression was used for model selection. All fixed explanatory variables and two-way interactions were fit and their significance tested using log-likelihood ratio tests (LRTs). The distribution fit to each model was chosen by considering the nature and dispersion of the data. To investigate significant associations between factor levels, post hoc comparisons were performed using Tukey tests.

Separate models of nightly and hourly abundance were fit to the data for each known *Anopheles* vector species (*An. balabacensis* and *An. flavirostris*) to investigate spatial and seasonal variation in their biting density between different ecotypes. The response variable for nightly models was the number of females collected per night, while the response variable for each hourly model was the number of females collected in each hourly period (18:00–19:00 to 05:00–06:00). Due to overdispersion in the mosquito count data, a negative binomial distribution was determined to be the best fit for all nightly and hourly models.

To investigate spatial variation in abundance, the site of collection was fit to the nightly and hourly models as a fixed factorial effect, with the unique mosquito collector identification (ID) and date of collection as random effects. Seasonal variation in nightly biting density was investigated with month of collection fit as a fixed continuous effect and as a quadratic variable to allow peaks in monthly biting density to be detected. To investigate hourly variations in biting density, the time at which the mosquito was collected was fit to the hourly model as a fixed continuous effect and as a quadratic variable with month of collection as a random effect.

To investigate variation in the biting density between collections with different human-baited and monkey-baited techniques, a model of nightly abundance was fit for each potential *Anopheles* vector species. The response variable for each model was the number of *Anopheles* females collected per night. Variations in abundance between traps were investigated with trap type fit to the model as a fixed factorial effect, while the collection station, date of collection and collector ID were included as random effects. A Poisson distribution was determined to be the best fit for all nightly models in this trap evaluation.

### Mosquito diversity

The species diversity indices were calculated for each trap type based on the *Anopheles* mosquito species collected. Species richness (R) is the total number of species collected by each trap type, accompanied by the Gini-Simpson diversity index (1–D), where

$$1 - D = 1 - \frac{{\sum n_{i} \left( {n_{i} - 1} \right)}}{{N\left( {N - 1} \right)}},$$

the 95% confidence limit of which is

$$\pm 2\sqrt {\frac{{\sum {\left( {\frac{{n_{i} }}{N}} \right)^{2} } - \left( {\sum {\left( {\frac{{n_{i} }}{N}} \right)^{2} } } \right)^{2} }}{{N\left( {N - 1} \right)}}},$$

where ${n}_{i}$ is the abundance of species $i$, and *N* is the total number of individuals in a sample.

## Results

### Longitudinal study of human-biting mosquitoes

In total, 4857 mosquitoes were collected across all sites over the 8 months of longitudinal sampling (Additional file 3: Table S1). Other *Anopheles* species were found in very low numbers, and only in the agricultural area and forest edge. A total of 124 female *Anopheles* mosquitoes belonging to nine species were obtained, of which *An. balabacensis* and *An. flavirostris* dominated (93.5% of all *Anopheles* females; Additional file 3: Table S2).

#### *Anopheles balabacensis* and *An. flavirostris* abundance across months

On account of their known role in transmission of malaria in the Philippines, further analysis was restricted to *An. balabacensis* and *An. flavirostris*. In general, the abundance of *An. balabacensis* was low (mean 0.34 to 1.20 per collection night). The best-fit model indicated that *An. balabacensis* density varied between sites (*χ*^2^ = 7.92, *df* = 1, *p* < 0.001), with biting density highest in the forest edge and lowest in the forest area (Additional file 4: Table S1). Comparison with the null model indicated that the best-fit model explained approximately 88.7% of the total deviance in the data.

**Fig. 3:**
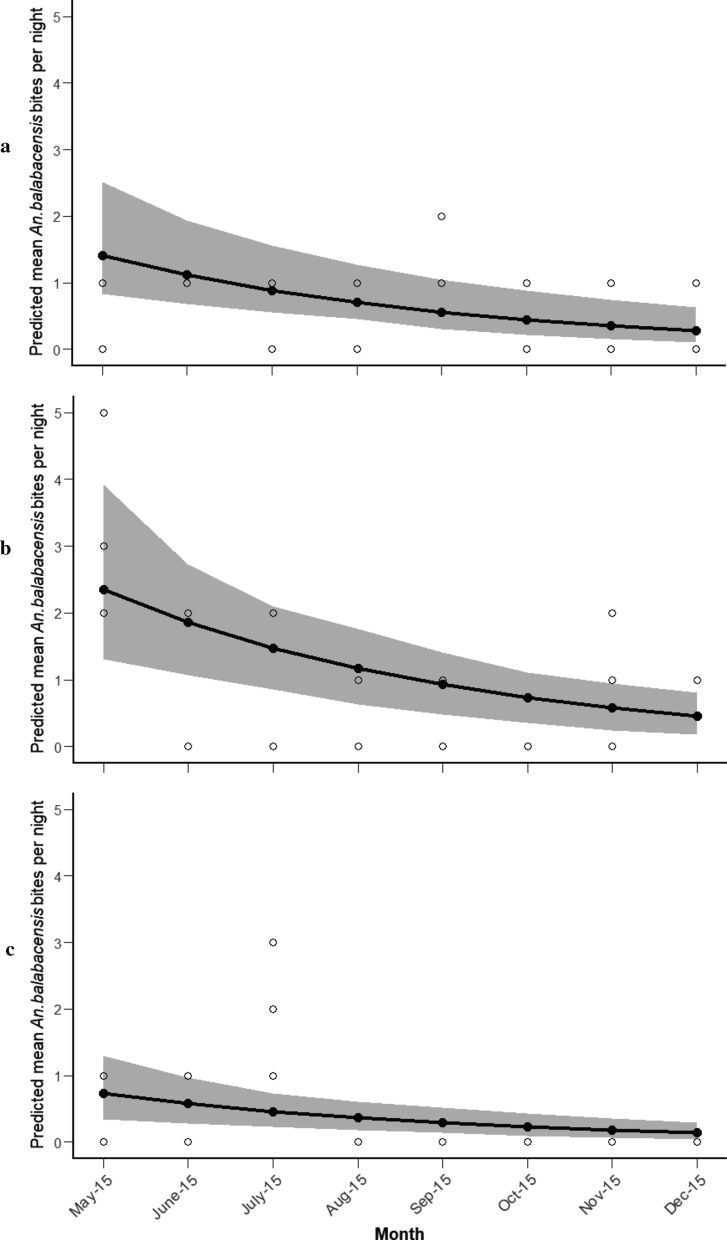
Predicted mean *An. balabacensis* bites per night from May to December 2015 (**a** agricultural area, **b** forest edge, **c** forest area; shaded area represents 95% CI, open circles represent observed values)

*Anopheles balabacensis* were collected in all months in the agricultural area and forest edge sites but were only observed in the forest area from May to July. There was no significant interaction between the month of collection and collection site (*χ*^2^ = 4.354, *df* = 2, *p* = 0.339), indicating a similar temporal pattern of abundance in all sites (Additional file 4: Table S1). The best-fit model indicated that abundance of *An. balabacensis* varied between months (*χ*^2^ = 10.68, *df* = 1, *p* = 0.01), with the highest biting density occurring in May, followed by a decline until December (Fig. 3).

The nightly density of *An. flavirostris* was also relatively low across the study area (mean 0 to 2 per collection night) with numbers ranging from 0 to 10 per night in the agricultural and forest edge sites, and none being collected in the forest site. The best fit model predicted that the abundance of *An. flavirostris* was higher in the agricultural area than the forest edge (Additional file 4: Table S2). Comparison with the null model indicated that the model explained approximately 78.4% of the total deviance in the data.

**Fig. 4:**
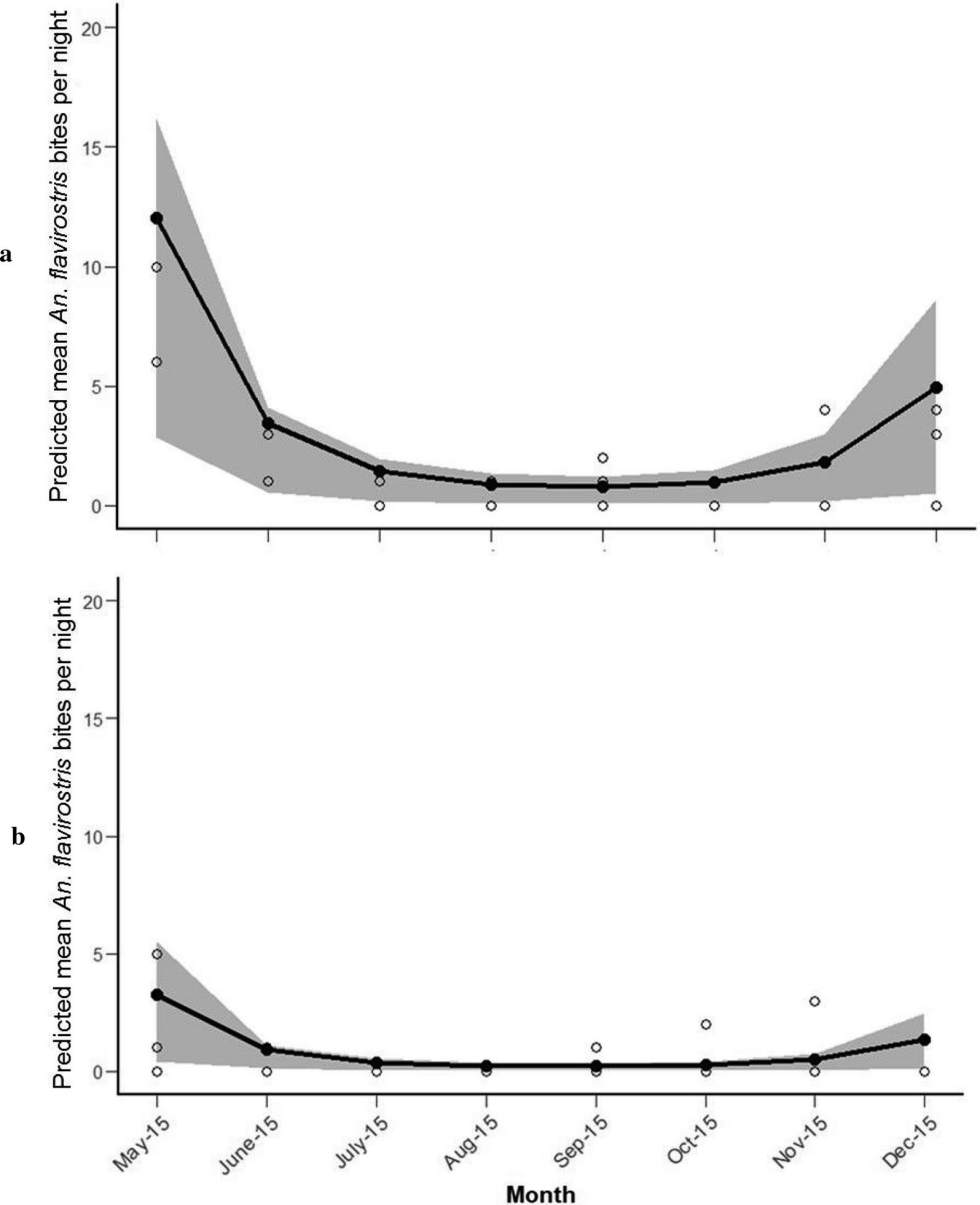
Predicted mean number of *An. flavirostris* bites per night from May to December 2015 (**a** agricultural area, **b** forest edge; shaded area represents 95% CI, open circles represent observed values)

*Anopheles flavirostris* was collected in the agricultural area across all months of collection; however, none were collected in the forest edge in July, August or December. There was no significant interaction between the month of collection and collection site (*χ*^2^ = 0.43, *df* = 1, *p* = 0.74), indicating similar seasonal patterns of *An. flavirostris* in the agricultural and forest edge areas (Additional file 4: Table S2). A quadratic association was observed between abundance of *An. flavirostris* and month of collection (*χ*^2^ = 15.248, *df* = 2, *p* < 0.001), characterized by peaks in abundance occurring in May and December (Fig. 4).

#### Hourly biting activity of *An. balabacensis* and *An. flavirostris*

Host-seeking *An. balabacensis* were collected throughout the sampling night (18:00–06:00) in both the agricultural and forest edge sites; however, no specimens were collected in the forest area before 19:00 or after 01:00. The number of *An. balabacensis* collected varied significantly throughout the night (*χ*^2^ = 34.93, *df* = 5, *p* < 0.001; Additional file 4: Table S3), with the model not detecting a difference in biting behavior between sites. Comparison with the null model indicated that the best-fit model explained approximately 92.6% of the total deviance in data.

**Fig. 5:**
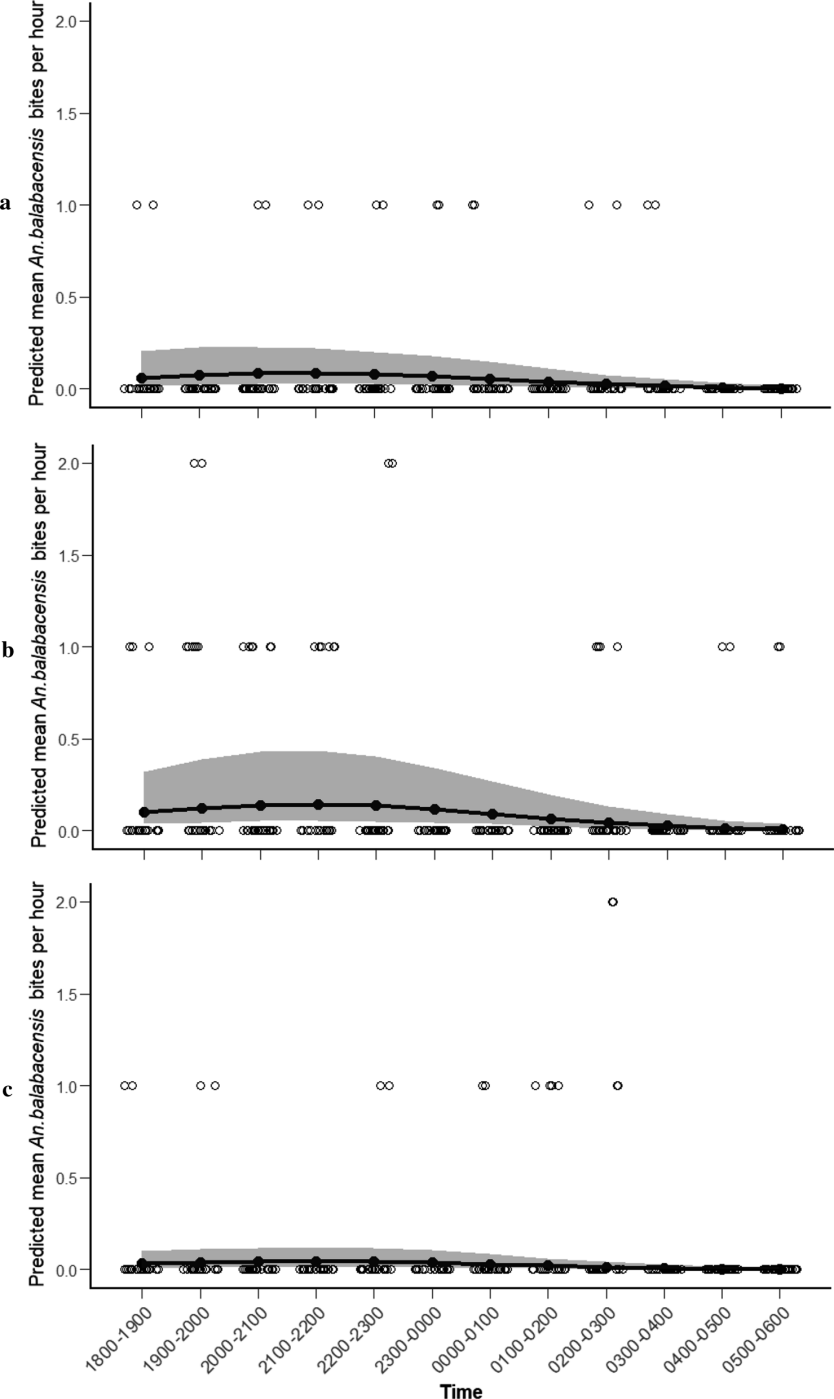
Predicted mean number of *An. balabacensis* bites per hour from 18:00 to 06:00 in each site (**a** agricultural area, **b** forest edge, **c** forest site; shaded area represents 95% CI, open circles represent observed values)

The number of *An. balabacensis* collected varied significantly over the course of a night (*χ*^2^ = 34.93, *df* = 5, *p* < 0.001), with the model predicting a single peak in abundance occurring between 21:00–22:00 followed by a gradual decline until dawn (Fig. 5). Approximately 60% of *An. balabacensis* bites occurred before 22:00 across all sites.

**Fig. 6:**
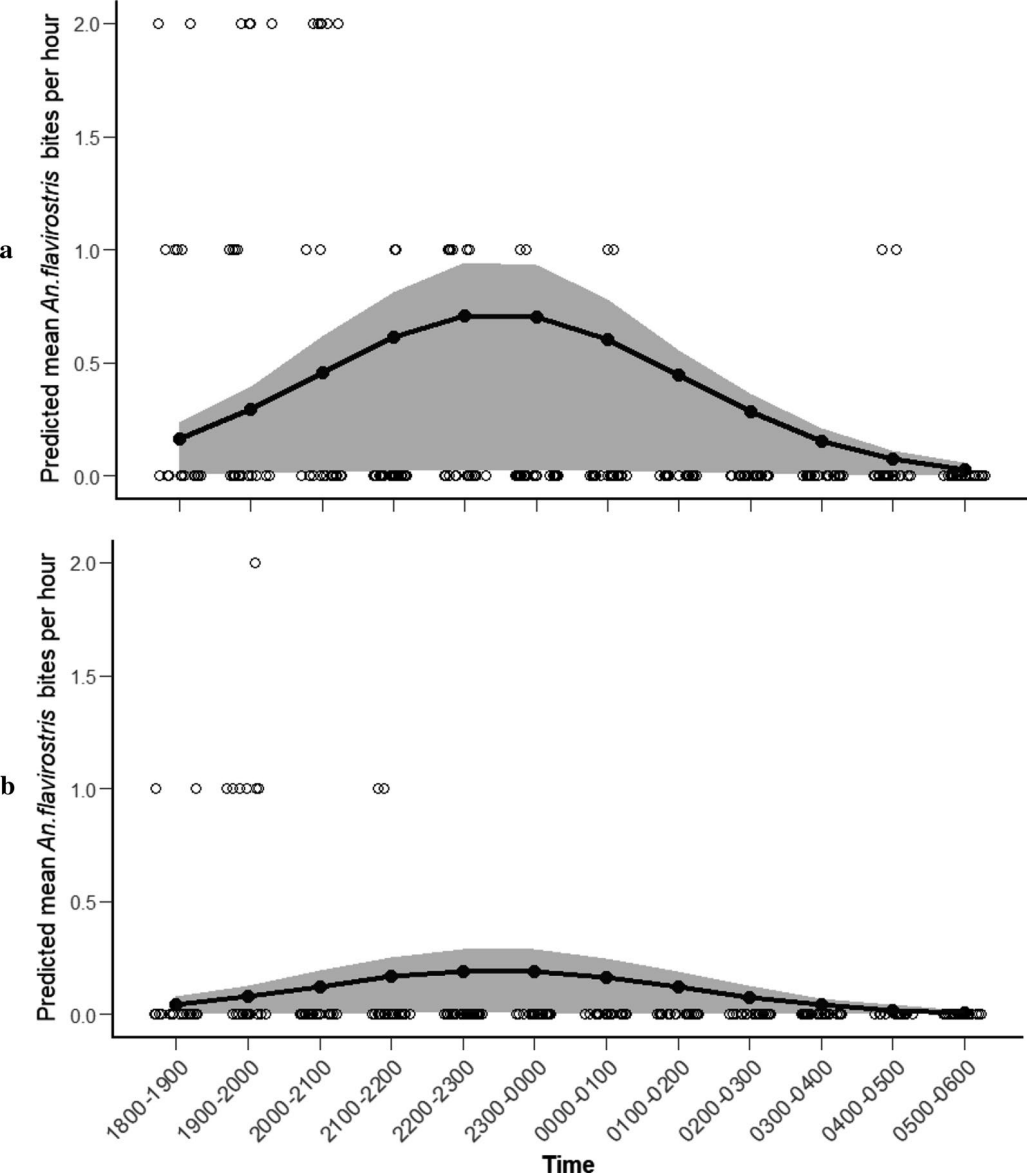
Predicted mean number of *An. flavirostris* bites per hour from 18:00 to 06:00 in each site (**a** agricultural area, **b** forest edge; shaded area represents 95% CI, open circles represent observed values)

*Anopheles flavirostris* was collected throughout the night in both the agricultural area and forest edge. The number of *An. flavirostris* collected varied significantly throughout the night (*χ*^2^ = 19.174, *df* = 2, *p* < 0.001; Additional file 4: Table S4). Comparison with the null model indicated that the best fit model explained approximately 87.5% of the total deviance in data. There was no significant interaction between *An. flavirostris* biting time and sample site (*χ*^2^ = 2.30, *df* = 1, *p* = 0.112) indicating the same hourly biting pattern in the agricultural area and forest edge. The model estimated peak abundance of *An. flavirostris* from 23:00 to 00:00 (Fig. 6), and only 33.86% of *An. flavirostris* bites occurring before 22:00.

### Comparison of human- and monkey-baited collections of *Anopheles* mosquitoes

#### Species composition of *Anopheles* mosquitoes collected in different traps

A total of 6591 mosquitoes were collected in all traps across 40 nights of outdoor collection, of which 3942 (59.81%) were females and subsequently identified to species level (Additional file 5: Table S1). Restricting analysis to the *Anopheles* genus, the majority of females were collected in MBT and the lowest numbers in HEN and MEN (Additional file 5: Table S2). The MBT collected the highest number of *Anopheles* species, with nine, while HEN, MEN and HLC caught eight, seven and four, respectively. The Gini-Simpson diversity index indicated that anopheline diversity was highest in the MEN trap and lowest in HLC collections.

#### Nightly abundance of *Anopheles* mosquitoes in each trap type

**Fig. 7:**
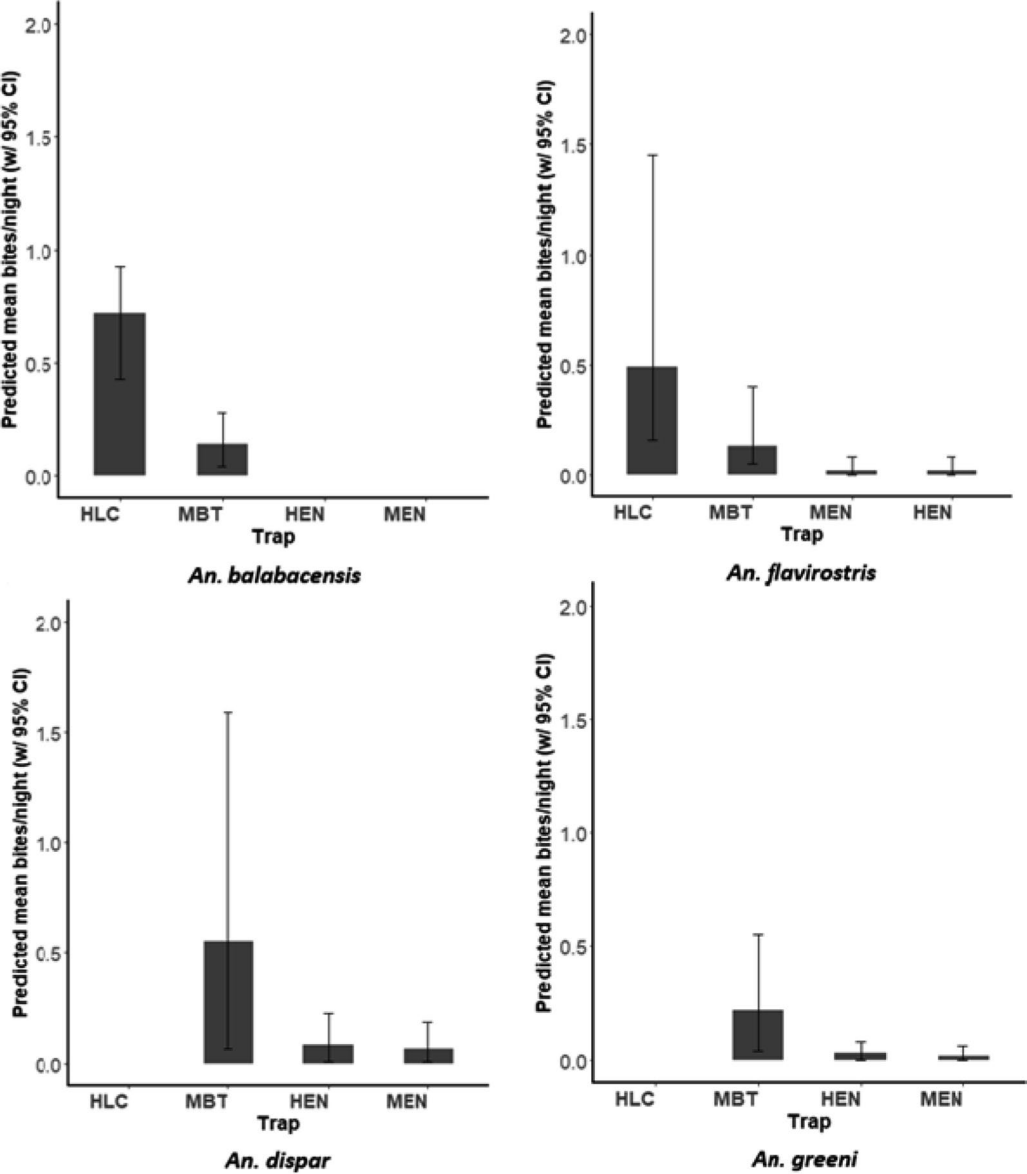
Predicted abundance of four *Anopheles* species (*An. balabacensis*, *An. flavirostris*, *An. dispar*, *An. greeni*) as sampled by different trapping methods (*HLC* human landing catch, *MBT* monkey-baited trap, *HEN* human-baited electrocuting net, *MEN* monkey-baited electrocuting net). No bar indicates no specimens of the species were collected in that trap

Statistical analysis of trap performance was conducted only for *An. balabacensis*, *An. flavirostris*, *An. dispar* and *An. greeni*, as the abundance of all other anopheline species was too low for robust analysis. For each of these species, statistical comparisons were made only between traps that collected at least one specimen. *Anopheles balabacensis* was collected in HLC and MBT at densities ranging from zero to three individuals per night, with none collected in the E-net traps. The mean abundance of *An. balabacensis* was approximately five times higher in HLC than in MBT collections (*χ*^2^ = 11.66, *df* = 1, *p* = 0.001, Fig. 7, Additional file 4: Table S5).

*Anopheles flavirostris* was collected in all four trap types. Biting densities of *An. flavirostris* ranged from zero to four bites per night and varied significantly between traps (*χ*^2^ = 36.93, *df* = 3, *p* = 0.001, Fig. 7). The HLC collected approximately 3.3 times more *An. flavirostris* than the MBT, with no difference between abundance in MBT, HEN and MEN (*p* > 0.05 in all cases; Additional file 5: Table S6).

*Anopheles dispar* and *An. greeni* were not collected by HLC. Biting densities of *An. dispar* varied significantly between trap types (*χ*^2^ = 34.56, *df* = 2, *p* = 0.001, Fig. 7), with abundance in MBT being 7.8 times higher than in HEN and MEN (Additional file 4: Table S7). *Anopheles greeni* densities varied significantly between trap types (MBT, HEN, MEN), with MBT yielding approximately 7.3 times more *An. greeni* than HEN or MEN (*χ*^2^ = 26.73, *df* = 2, *p* = 0.001; Additional file 4: Table S8, Fig. 7). However, there was no significant difference between collections with HEN and MEN (*p* = 0.89).

### Molecular detection of *Plasmodium *in *Anopheles* mosquitoes

All female *Anopheles* mosquitoes (*n* = 357) collected during the study were tested for the presence of malaria parasites. A total of 120 pooled samples underwent a first round of nested PCR and all were all negative for *Plasmodium* parasites.

## Discussion

To better understand the low incidence of *P. knowlesi* in Palawan despite its close proximity to a major focus of infection in nearby Sabah, Malaysian Borneo, here we characterized the ecology and biting behavior of potential *Anopheles* vectors across three ecotypes representative of land use. Two known malaria vectors, *An. balabacensis* (the vector of *P. knowlesi* in Sabah) and *An. flavirostris*, were detected in longitudinal surveillance in Palawan, representing 44% and 49% of all anophelines, respectively. However, mean nightly human-biting densities were low, ranging from 0.34 to 1.20 for *An. balabacensis* and 0 to 2 for *An. flavirostris*. A substantial proportion of *Anopheles* bites occurred before 10 pm, a time when residents in Palawan would typically be active and unprotected by insecticide-treated nets. No *Plasmodium-*infected mosquitos were found, though the small number collected meant that detection power was limited. Sampling with human- and macaque-baited traps indicated that both vector species are attracted to each host type, and could thus serve as bridge vectors for *P. knowlesi.* In summary, while potential vectors of *P. knowlesi* are present in Palawan, their comparatively low densities and infection rates indicate that human exposure to *P. knowlesi* is considerably lower in this setting than in nearby Sabah, where this parasite is the primary cause of malaria in humans.

The outdoor biting densities of potential *P. knowlesi* vectors *Anopheles* in Palawan were approximately seven times lower than that found in recent studies in northern Sabah (e.g. *An. balabacensis* ranging from 1.81 to 7.84 bites per night) [19, 27]. More recent, broader sampling across Sabah state revealed substantial geospatial variation in *An. balabacensis* biting densities, confirming that *An. balabacensis* abundance is highly heterogeneous even across even short distances [26]. The relative abundance of *An. balabacensis* observed in the current study (44% of all anophelines) falls in the middle range of what has been previously reported in other settings in Malaysian Borneo (e.g. from 15% [26], 40% [19] and 95% [27]).

No *Anopheles* specimen collected in this study tested positive for *Plasmodium,* thus definitive incrimination of the contemporary *P. knowlesi* vector in Palawan was not possible. For comparison, in Sabah the Plasmodium infection rates (all species) in *An. balabacensis* ranged from 1.45–3% [26, 27, 37–39], with *P. knowlesi*-specific rates ranging from 0–3% [26, 27, 37–39]. Thus even in areas of high *P. knowlesi* transmission to humans, infection rates in vectors are relatively low. Failure to detect *P. knowlesi* in vectors collected here should not be interpreted as evidence of an absence of transmission. The relatively small number (*n* = 357) of *Anopheles* collected may have insufficient to detect infection, especially if transmission was occurring at low levels. A much larger sample may be required to accurately estimate the prevalence of *P. knowlesi* infection in *Anopheles* populations in Palawan. Although not confirmed in this study, we hypothesize that *An. balabacensis* remains the most likely *P. knowlesi* vector in Palawan based on previous work [28].

The biting density of *An. balabacensis* and *An. flavirostris* varied between collection sites. *Anopheles balabacensis* was more abundant in the forest edge than forest site, with density in the agricultural site being statistically in distinguishable from either forest site. Previous focal sampling in northern Sabah showed that *An. balabacensis* was also more abundant at forest edges than in human settlements [21], and in farm and forest than in peri-domestic habitats [26]. Thus, our findings are consistent in highlighting the suitability of forest edge habitats for *An. balabacensis*. In contrast, *An. flavirostris* density was significantly higher in the agricultural than the forest edge habitat, with no individuals collected in the forest site in contrast to previous studies in Palawan [40, 41]. The absence of *An. flavirostris* in our forest site may be due to site-specific effects, with broader sampling over a range of forest sites required to confirm habitat associations.

There was evidence of seasonality in both *An. balabacensis* and *An. flavirostris* populations in Palawan, although the pattern varied somewhat between vector species. The abundance of *An. balabacensis* was highest in May, followed by a gradual decrease through the remaining months of surveillance until December. In contrast, longitudinal sampling in Sabah [27] revealed month-to-month variation in *An. balabacensis* but no consistent seasonal trend between sites. Seasonality in *An. flavirostris* was characterized by peaks in biting density in May and December, with a decline in density during the intermediate months. As vectors were only sampled from May to December here, it is possible that the annual peak in *An. balabacensis* or *An. flavirostris* lies outside the sampling period investigated. However this is unlikely as rainfall and malaria transmission are strongly seasonal in Palawan, with the peak period of rains and malaria transmission (June–August/September) falling within the sampling period [42]. Notably, the peaks in *An. balabacensis* (May) and *An. flavirostris* (May and December) occurred outside the main period of rains in Puerto Princesa City.

The trap evaluation study revealed substantial differences in *Anopheles* species composition between trapping methods. *Anopheles balabacensis* and *An. flavirostris* were most abundant in HLC whereas *An. dispar* and *An. greeni* were dominant in MBTs. The *P. knowlesi* vector *An. balabacensis* was five times more abundant in HLCs than in MBTs. This difference may reflect a preference for humans over macaques for *An. balabacensis*; however, results from HLC and MBT may not be directly comparable due to non-host-related differences in trapping methods. Nevertheless, these results are consistent with a similar study in Sabah, where *An. balabacensis* was collected more frequently with HLC than with MBT [19]. To our knowledge, this is the first direct comparison of *An. flavirostris* host-seeking on human and macaque hosts. Similar to *An. balabacensis*, *An. flavirostris* was more abundant (~ 3.3 times) in HLC than MBT collections. *Anopheles flavirostris* has been previously described as zoophilic based on comparisons between human- and water buffalo-baited collections [41, 43, 44]. The other two *Anopheles* species that were common in MBTs, *An. dispar* and *An. greeni*, are indigenous to the Philippines. There is no definitive evidence that these species are involved in human malaria transmission [30]; but their apparent preference for macaques over humans here indicates that they have potential to act as vectors for simian malaria.

In comparison to the HLC and MBT methods, the E-nets used in this study (HEN and MEN) performed relatively poorly for anopheline surveillance. Whilst almost all mosquitoes in HLCs were *An. balabacensis* and *An. flavirostris*, HEN collected no *An. balabacensis* and only one *An. flavirostris*. Similarly, the MEN collected fewer anophelines than the MBT, although numbers were sufficient to give an adequate representation of species diversity. The poor sampling efficiency of the E-nets and lack of difference in species composition between HEN and MEN compared to that between HLC and MBT collections is consistent with previous evaluations in Sabah [19]. The E-net traps’ poorer performance relative to the HLC and MBT may be due to the design of the current prototype, where host odors are pumped from the tent along the length of PVC pipe to the electrified grid. The long (6 m) pipe or relatively fast movement of air may reduce or dilute the quantity or quality of the odor cues needed by mosquitoes to identify and locate their preferred host species. In summary, these findings indicate that the E-net traps used here do not provide an appropriate representation of the standard HLC and MBT methods.

Current malaria control strategies in Palawan rely on the use of LLINs and IRS [1]. As these interventions primarily target indoor biting mosquitoes, they are likely insufficient for protection against the outdoor, early-biting vectors of *P. knowlesi*. For example, in outdoor collections here, 60.37% of biting by *An. balabacensis* and 33.68% by *An. flavirostris* occurred between 18:00 and 22:00; a period in the evening when many people in rural communities in Palawan would still be outdoors. These findings are consistent with previous investigations in Sabah, where a large proportion of outdoor *An. balabacensis* bites occurred outdoors in the early evening, with almost no evidence of indoor biting [20, 37]. Clearly, additional vector control strategies that can protect people outside of homes are needed to reduce the risk of *P. knowlesi* exposure in Palawan and other settings where it is emerging.

## Conclusions

The monkey malaria *P. knowlesi* is now the primary cause of human malaria in Malaysian Borneo; however only sporadic human cases have been reported in the nearby island of Palawan. By investigating the ecology and behavior of potential *P. knowlesi* vectors in Palawan, this study indicates that this disparity may be due to the relatively lower density and infection rates in mosquitoes even though known vector species are present. The reason for lower vector densities in this setting is unknown, but may relate to differences in land use and fragmentation between Palawan and northern Sabah.

While the risk of *P. knowlesi* spillover to humans in Palawan is low at present, it could increase with land use or other socioecological changes. To mitigate against the risk of *P. knowlesi* and other malaria species transmitted by exophilic vectors, control strategies in Palawan may need to be expanded to incorporate methods that protect people when they are outdoors.

## Supplementary Information

**Additional file 1. **Ethical considerations for the use of non-human primates.

**Additional file 2. **Detail of PCR primers used for detection of malaria parasite species in *Anopheles* mosquito specimens.

**Additional file 3: Table S1.** Summary of total number of mosquitoes caught in the different collection sites in the longitudinal study. **Table S2.** Female *Anopheles* species collected in each sampling site in Puerto Princesa City, Palawan from May to December 2015.

**Additional file 4: Table S1.** Summary of modeled coefficients for the nightly abundance of *An. Balabacensis* collected in each site in the longitudinal study from May to December 2015 (a – statistical difference relative to zero, b – statistical difference relative to reference category, * – indicates statistically significant difference). **Table S2.** Summary of modeled coefficients for the nightly abundance of *An. flavirostris* collected in the longitudinal study from May to December 2015 (a – statistical difference relative to zero, b – statistical difference relative to reference category, * – indicates statistically significant difference). **Table S3.** Summary of modeled coefficients for the hourly abundance of *An. balabacensis* collected in longitudinal study from May to December 2015 (a – statistical difference relative to zero, b – statistical difference relative to reference category, * – indicates statistically significant difference). **Table S4.** Summary of modeled coefficients for the hourly abundance of *An. flavirostris* collected in longitudinal study from May to December 2015 (a – statistical difference relative to zero, b – statistical difference relative to reference category, * – indicates statistically significant difference). **Table S5.** Summary of modeled coefficients for the nightly abundance of *An. balabacensis* collected in each trap (HLC – human landing catch, MBT – monkey-baited trap, HEN – human-baited electrocuting net, MEN – monkey-baited electrocuting net, a – statistical difference relative to zero, b – statistical difference relative to reference category, * – indicates statistically significant difference). **Table S6.** Summary of modeled coefficients for the nightly abundance of *An. flavirostris* collected in each trap (HLC – human landing catch, MBT – monkey-baited trap, HEN – human-baited electrocuting net, MEN – monkey-baited electrocuting net, a – statistical difference relative to zero, b – statistical difference relative to reference category, * – indicates statistically significant difference). **Table S7.** Summary of modeled coefficients for the nightly abundance of *An. dispar* collected in each trap (HLC – human landing catch, MBT – monkey-baited trap, HEN – human-baited electrocuting net, MEN – monkey-baited electrocuting net, a – statistical difference relative to zero, b – statistical difference relative to reference category, * – indicates statistically significant difference). **Table S8.** Summary of modeled coefficients for the nightly abundance of *An. greeni* collected in each trap (HLC – human landing catch, MBT – monkey-baited trap, HEN – human-baited electrocuting net, MEN – monkey-baited electrocuting net, a – statistical difference relative to zero, b – statistical difference relative to reference category, * – indicates statistically significant difference).

**Additional file 5: Table S1.** Summary of total number of mosquitoes caught using the different trapping techniques in the trap comparison study. **Table S2.** Total number of female *Anopheles* mosquitoes collected in each trap and associated diversity indices (HLC – human landing catch, MBT – monkey-baited trap, HEN – human-baited electrocuting net, MEN – monkey-baited electrocuting net).

## Data Availability

Summary data supporting the conclusions of this article are included within the article and it’s additional files. Raw data can be made available through request to the Director of RITM, Philippines.

## Abbreviations

GLMM: Generalized linear mixed models
HEN: Human-baited electrocuting net
HLC: Human landing catch
IRS: Indoor residual spraying
LLIN: Long-lasting insecticidal net
MBT: Monkey-baited trap
MEN: Monkey-baited electrocuting net
PCR: Polymerase chain reaction
RITM: Research Institute for Tropical Medicine
