# Supplementary material for: The seasonal dynamics and biting behavior of potential Anopheles vectors of Plasmodium knowlesi in Palawan, Philippines
Source: Parasit Vectors. 2021 Jul 7;14:357. doi: 10.1186/s13071-021-04853-9 (PMC8261946; doi:10.1186/s13071-021-04853-9)
Supplement: Supplementary file 1 — Additional file 1. Ethical considerations for the use of non-human primates. [file 13071_2021_4853_MOESM1_ESM.docx]

**Additional File 1. Ethical considerations for the use of non-human primates**

Domestic/captive macaques used in the experiments were obtained from Palawan Wildlife Rescue and Conservation Center (PWRCC) in Puerto Princesa City. All husbandry and veterinary care of macaques was provided by a trained animal technician and a veterinarian from PWRCC. Prior to involvement in the study, individual macaques underwent a veterinary assessment, including screening for infection with *P. knowlesi*, hepatitis A, B and C, and tuberculosis, and behavioral observations to assess compatibility with other macaques. Their health and welfare were monitored continually by the animal technician who was stationed in rotation with the macaques throughout the study. A veterinary assessment of all macaques was conducted every month to monitor their general health conditions.

Following the Animal (Scientific Procedures) Act 1986 Code of Practice for the Housing and Care of Animals Used in Scientific Procedures [1] and guidelines set down by the National Centre for the Replacement, Refinement and Reductions of Animals in Research [2], macaques were held in floor-to-ceiling cages with a height of 2.0 m and minimum enclosure size of 2m^2^ to give each animal a minimum volume of 1.8 m^3^ as stipulated in the Act [2]. Cages were fitted with an elevated platform, to provide roosting space, and enrichment items, including rope, bedding, balls and chew toys [3]. Water was provided freely and diet items hidden around the enclosure and inside toys, as well as being left unpeeled/unshelled to provide stimulation and allow macaques to express natural foraging behavior [2].

As only three macaques were available for use in this study, macaques were rotated between collection stations each night to meet the requirements of ethics approval. Each macaque was used for a maximum of 10 mosquito collection nights per month. Two macaques participated in each night of the study while the remaining macaque remained in their cage but was not involved in mosquito collections. Transfer of macaques between mosquito collection stations was done during the early morning hours and was done by the animal technician with the assistance of the research assistants and field workers. Close monitoring was done on the macaques after each transfer to check any changes in their behavior.

**References**

[1] Animals (Scientific Procedures) Act 1986. Available at: http://www.legislation.gov.uk/ukpga/1986/14/contents (Accessed 31st October 2016).

[2] NC3Rs. Guidelines – Primate accommodation, care and use. London: NC3Rs. 2006.

[3] Waitt CD, Honness PE, Bushmitz M. Creating housing to meet the behavioural needs of Long-tailed macaques. Primate Newsletter. 2008;47(4):1-5.
