# Supplementary material for: The seasonal dynamics and biting behavior of potential Anopheles vectors of Plasmodium knowlesi in Palawan, Philippines
Source: Parasit Vectors. 2021 Jul 7;14:357. doi: 10.1186/s13071-021-04853-9 (PMC8261946; doi:10.1186/s13071-021-04853-9)
Supplement: Supplementary file 2 — Additional file 2. Detail of PCR primers used for detection of malaria parasite species in Anopheles mosquito specimens. [file 13071_2021_4853_MOESM2_ESM.docx]

**Additional File 2. Detail of PCR primers used for detection of malaria parasite species in Anopheles mosquito specimens**

| Target | Genus/species | Primer name | Sequence (5’ - 3’) | Annealing temp. (^o^C) | Size of PCR product (bp) | Reference |
| --- | --- | --- | --- | --- | --- | --- |
| SSU-rRNA | *Plasmodium* genus | rPLU1 | TCAAAGATTAAGCCATGCAAGTGA | 55 | 1640 | ^[1]^ |
|  |  | rPLU5 | CCTGTTGTTGCCTTAAACTCC |  |  | ^[1]^ |
|  |  | rPLU3 | TTTTTATAAGGATAACTACGGAAAAGCTGT | 62 | 240 | ^[1]^ |
|  |  | rPLU4 | TACCCGTCATAGCCATGTTAGGCCAATACC |  |  | ^[1]^ |
| SSU-rRNA | *P. coatneyi* | PctF1 | CGCTTTTAGCTTAAATCCACATAACAGAC | 62 | 504 | ^[2]^ |
|  |  | PctR1 | GAGTCCTAACCCCGAAGGGAAAGG |  |  | ^[2]^ |
|  | *P. inui* | PinF2 | CGTATCGACTTTGTGGCATTTTTCTAC | 60 | 479 | ^[2]^ |
|  |  | INAR3 | GCAATCTAAGAGTTTTAACTCCTC |  |  | ^[2]^ |
|  | *P. fieldi* | PfldF1 | GGTCTTTTTTTTGCTTCGGTAATTA | 66 | 421 | ^[2]^ |
|  |  | PfldR2 | AGGCACTGAAGGAAGCAATCTAAGAGTTTC |  |  | ^[2]^ |
|  | *P. cynomolgi* | CY2F | GATTTGCTAAATTGCGGTCG | 60 | 137 | ^[2]^ |
|  |  | CY4R | CGGTATGATAAGCCAGGGAAGT |  |  | ^[2]^ |
|  | *P. knowlesi* | PkF1140 | GATTCATCTATTAAAAATTTGCTTC | 50 | 424 | ^[3]^ |
|  |  | PkR1550 | GAGTTCTAATCTCCGGAGAGAAAAGA |  |  | ^[3]^ |
|  | *P. falciparum* | NewPLFshort | CTATCAGCTTTTGATGTTAG | 53 | 370 | ^[4]^ |
|  |  | FARshort | GTTCCCCTAGAATAGTTACA |  |  | ^[4]^ |
|  | *P. vivax* | NewPLFshort | CTATCAGCTTTTGATGTTAG | 53 | 476 | ^[4]^ |
|  |  | VIRshort | AAGGACTTCCAAGCC |  |  | ^[4]^ |
|  | *P. malariae* | NewPLFshort | CTATCAGCTTTTGATGTTAG | 53 | 241 | ^[4]^ |
|  |  | MARshort | TCCAATTGCCTTCTG |  |  | ^[4]^ |
|  | *P. ovale* | NewPLFshort | CTATCAGCTTTTGATGTTAG | 53 | 407 | ^[4]^ |
|  |  | OVRshort | AGGAATGCAAAGARCAG |  |  | ^[4]^ |

^[1]^Singh B, Bobogare A, Cox-Singh J, Snounou G, Abdullah MS, Rahman HA. A genus- and species-specific nested polymerase chain reaction malaria detection assay for epidemiologic studies. Am J Trop Med Hyg. 1999;60(4):687-692.

^[2]^Lee KS, Divis PCS, Zakaria SK, Matusop A, Julin RA, Conway DJ et al. *Plasmodium knowlesi*: Reservoir hosts and tracking the emergence in humans and macaques. Plos Pathog. 2011;7:4.

^[3]^Imwong M, Tanomsing N, Pukrittayakamee S, Day, NPJ, White NJ, Snounou G. Spurious amplification of a *Plasmodium vivax* small-subunit RNA gene by use of primers currently used to detect *P. knowlesi.* J Clin Microbiol. 2009;47(12):4173-4175.

^[4]^Ta TH, Hisam S, Lanza M, Jiram AI, Ismail NP, Rubio JM. First case of a naturally acquired human infection with *Plasmodium cynomolgi*. Malaria J. 2014;13:68.
