# Supplementary material for: The seasonal dynamics and biting behavior of potential Anopheles vectors of Plasmodium knowlesi in Palawan, Philippines
Source: Parasit Vectors. 2021 Jul 7;14:357. doi: 10.1186/s13071-021-04853-9 (PMC8261946; doi:10.1186/s13071-021-04853-9)
Supplement: Supplementary file 3 — Additional file 3: Table S1. Summary of total number of mosquitoes caught in the different collection sites in the longitudinal study. Table S2. Female Anopheles species collected in each sampling site in Puerto Princesa City, Palawan from May to December 2015. [file 13071_2021_4853_MOESM3_ESM.docx]

**Additional File 3.**

Table S1. Summary of total number of mosquitoes caught in the different collection sites in the longitudinal study

| **Species** | **Collection Site** | | | **Female** | **Male** | **Total** | **% Composition** |
| --- | --- | --- | --- | --- | --- | --- | --- |
|  | **Agricultural Area** | **Forest Edge** | **Forest Area** |  |  |  |  |
| *An. balabacensis* | 17 | 29 | 9 | 55 | 0 | 55 | 1.13 |
| *An. flavirostris* | 48 | 13 | 0 | 61 | 0 | 61 | 1.26 |
| *An. dispar* | 1 | 0 | 0 | 1 | 0 | 1 | 0.02 |
| *An. annularis* | 1 | 0 | 0 | 1 | 0 | 1 | 0.02 |
| *An. franciscoi* | 0 | 1 | 0 | 1 | 0 | 1 | 0.02 |
| *An. ludlowae* | 1 | 0 | 0 | 1 | 0 | 1 | 0.02 |
| *An. pseudobarbirostris* | 1 | 0 | 0 | 1 | 0 | 1 | 0.02 |
| *An. subpictus* | 1 | 1 | 0 | 2 | 0 | 2 | 0.04 |
| *An. vagus* | 2 | 0 | 0 | 1 | 1 | 2 | 0.04 |
| *Ae. albopictus* | 45 | 20 | 26 | 89 | 2 | 91 | 1.87 |
| *Ae. flavipennis* | 1 | 31 | 21 | 39 | 14 | 53 | 1.09 |
| *Ae. poicilius* | 1 | 5 | 0 | 6 | 0 | 6 | 0.12 |
| *Ae. vigilax* | 8 | 10 | 101 | 119 | 0 | 119 | 2.45 |
| *Ar. kesseli* | 1 | 0 | 0 | 1 | 0 | 1 | 0.02 |
| *Ar. malayi* | 21 | 10 | 6 | 37 | 0 | 37 | 0.76 |
| *Ar. subalbatus* | 768 | 51 | 52 | 869 | 2 | 871 | 17.93 |
| *Ar. theobaldi* | 1 | 0 | 0 | 1 | 0 | 1 | 0.02 |
| *Cx. bitaeniorhynchus* | 1 | 1 | 0 | 2 | 0 | 2 | 0.04 |
| *Cx. fuscocephala* | 0 | 5 | 0 | 2 | 3 | 5 | 0.10 |
| *Cx. gelidus* | 1 | 0 | 0 | 1 | 0 | 1 | 0.02 |
| *Cx. hutchinsoni* | 5 | 11 | 0 | 3 | 13 | 16 | 0.33 |
| *Cx. jacksoni* | 1 | 0 | 0 | 0 | 1 | 1 | 0.02 |
| *Cx. quinquefasciatus* | 2 | 0 | 0 | 2 | 0 | 2 | 0.04 |
| *Cx. sinensis* | 0 | 3 | 0 | 3 | 0 | 3 | 0.06 |
| *Cx. tritaeniorhynchus* | 1 | 0 | 0 | 1 | 0 | 1 | 0.02 |
| *Cx. vishnui* | 1,480 | 734 | 772 | 2,983 | 3 | 2,986 | 61.48 |
| *Do. ganapathi* | 3 | 44 | 152 | 199 | 0 | 199 | 4.10 |
| *Lo. fumida* | 0 | 0 | 2 | 2 | 0 | 2 | 0.04 |
| *Lt. halifaxii* | 1 | 0 | 0 | 1 | 0 | 1 | 0.02 |
| *Tr. aeneus* | 2 | 4 | 0 | 2 | 4 | 6 | 0.12 |
| *Ur. hebes* | 0 | 1 | 0 | 1 | 0 | 1 | 0.02 |
| *Ur. metatarsata* | 9 | 1 | 2 | 5 | 7 | 12 | 0.25 |
| *Downsiomyia* sp. | 7 | 65 | 189 | 261 | 0 | 261 | 5.37 |
| *Lutzia* sp. | 0 | 1 | 0 | 1 | 0 | 1 | 0.02 |
| *Mimomyia* sp. | 0 | 1 | 0 | 1 | 0 | 1 | 0.02 |
| *Tripteroides* sp. | 1 | 2 | 2 | 5 | 0 | 5 | 0.10 |
| *Uranotaenia* sp. | 20 | 23 | 0 | 28 | 15 | 43 | 0.89 |
| *Verralina* sp. | 0 | 1 | 0 | 0 | 1 | 1 | 0.02 |
| Unidentified | 3 | 0 | 0 | 2 | 1 | 3 | 0.06 |
| **TOTAL** | **2,455** | **1,068** | **1,334** | **4,790** | **67** | **4,857** | **100.00** |

Table S2. Female *Anopheles* species collected in each sampling site in Puerto Princesa City, Palawan from May to December 2015

| **Mosquito Species** | **Agricultural Area** | **Forest-edge** | **Forest Area** | **Total (%)** |
| --- | --- | --- | --- | --- |
| *An. balabacensis* | 17 | 29 | 9 | 55 (44.35) |
| *An. flavirostris* | 48 | 13 | 0 | 61 (49.19) |
| *An. dispar* | 1 | 0 | 0 | 1 (0.81) |
| *An. annularis* | 1 | 0 | 0 | 1 (0.81) |
| *An. franciscoi* | 0 | 1 | 0 | 1 (0.81) |
| *An. ludlowae* | 1 | 0 | 0 | 1 (0.81) |
| *An. pseudobarboristris* | 1 | 0 | 0 | 1 (0.81) |
| *An. subpictus* | 1 | 1 | 0 | 2 (1.61) |
| *An. vagus* | 1 | 0 | 0 | 1 (0.81) |
| **Total** | **71** | **44** | **9** | **124(100)** |
