# Supplementary material for: The seasonal dynamics and biting behavior of potential Anopheles vectors of Plasmodium knowlesi in Palawan, Philippines
Source: Parasit Vectors. 2021 Jul 7;14:357. doi: 10.1186/s13071-021-04853-9 (PMC8261946; doi:10.1186/s13071-021-04853-9)
Supplement: Supplementary file 4 — Additional file 4: Table S1. Summary of modeled coefficients for the nightly abundance of An. Balabacensis collected in each site in the longitudinal study from May to December 2015 (a – statistical difference relative to zero, b – statistical difference relative to reference category, * – indicates statistically significant difference). Table S2. Summary of modeled coefficients for the nightly abundance of An. flavirostris collected in the longitudinal study from May to December 2015 (a – statistical difference relative to zero, b – statistical difference relative to reference category, * – indicates statistically significant difference). Table S3. Summary of modeled coefficients for the hourly abundance of An. balabacensis collected in longitudinal study from May to December 2015 (a – statistical difference relative to zero, b – statistical difference relative to reference category, * – indicates statistically significant difference). Table S4. Summary of modeled coefficients for the hourly abundance of An. flavirostris collected in longitudinal study from May to December 2015 (a – statistical difference relative to zero, b – statistical difference relative to reference category, * – indicates statistically significant difference). Table S5. Summary of modeled coefficients for the nightly abundance of An. balabacensis collected in each trap (HLC – human landing catch, MBT – monkey-baited trap, HEN – human-baited electrocuting net, MEN – monkey-baited electrocuting net, a – statistical difference relative to zero, b – statistical difference relative to reference category, * – indicates statistically significant difference). Table S6. Summary of modeled coefficients for the nightly abundance of An. flavirostris collected in each trap (HLC – human landing catch, MBT – monkey-baited trap, HEN – human-baited electrocuting net, MEN – monkey-baited electrocuting net, a – statistical difference relative to zero, b – statistical difference relative to reference category, * [file 13071_2021_4853_MOESM4_ESM.docx]

**Additional File 4**

Table S1. Summary of modelled coefficients for the nightly abundance of *An. balabacensis*

collected in each site in the longitudinal study from May to December 2015 (a – statistical

difference relative to zero, b – statistical difference relative to reference category, * – indicates

statistically significant difference)

| **Variable** | **Type** | **Levels** | **Coefficient** | **SE** | **p value** |
| --- | --- | --- | --- | --- | --- |
| **Site** | Factorial | Agricultural area (reference) | 0.252 | 0.665 | **0.09 (a)** |
|  |  | Forest edge | 0.610 | 0.785 | **0.12 (b)** |
|  |  | Forest area | 1.265 | 0.613 | **0.11 (b)** |
| **Month** | Continuous | N/A | -0.270 | 0.301 | **0.01*** |
| **Month^2** | Continuous | N/A | -0.386 | 0.224 | **0.004*** |
| **Site*Month** | Factorial: Continuous | Forest edge: Month | -0.386 | 0.224 | **0.339** |
|  |  | Forest area: Month | -0.203 | 0.139 | **0.339** |

Table S2. Summary of modelled coefficients for the nightly abundance of *An. flavirostris*

collected in the longitudinal study from May to December 2015 (a – statistical difference relative

to zero, b – statistical difference relative to reference category, * – indicates statistically significant

difference)

| **Variable** | **Type** | **Levels** | **Coefficient** | **SE** | **p value** |
| --- | --- | --- | --- | --- | --- |
| **Site** | Factorial | Forest area (reference) | 3.381 | 0.724 | **<0.001* (a)** |
|  |  | Forest edge | -1.307 | 0.343 | **<0.001* (b)** |
| **Month** | Continuous | N/A | -1.812 | 0.436 | **<0.001* (b)** |
| **Month^2** | Continuous | N/A | 0.188 | 0.048 | **<0.001*** |
| **Site*Month** | Factorial: Continuous | Forest edge: Month | 0.091 | 0.118 | **0.745** |

Table S3. Summary of modelled coefficients for the hourly abundance of *An. balabacensis* collected in longitudinal study from May to December 2015 (a – statistical difference relative to zero, b – statistical difference relative to reference category, * – indicates statistically significant difference)

| **Variable** | **Type** | **Levels** | **Coefficient** | **SE** | **p value** |
| --- | --- | --- | --- | --- | --- |
| **Site** | Factorial | Agricultural area (reference) | -3.041 | 0.557 | **<0.001* (a)** |
|  |  | Forest edge | 0.517 | 0.315 | **0.12 (b)** |
|  |  | Forest area | -0.652 | 0.419 | **0.10 (b)** |
| **Time** | Continuous | N/A | 0.335 | 0.199 | **<0.001*** |
| **Time^2** | Continuous | N/A | -0.043 | 0.017 | **0.007*** |
| **Site*Time** | Factorial: Continuous | Forest edge: Time | -0.076 | 0.119 | **0.494** |
|  |  | Forest area: Time | -0.199 | 0.175 | **0.494** |

Table S4. Summary of modelled coefficients for the hourly abundance of *An. flavirostris* collected in longitudinal study from May to December 2015 (a – statistical difference relative to zero, b – statistical difference relative to reference category, * – indicates statistically significant difference)

| **Variable** | **Type** | **Levels** | **Coefficient** | **SE** | **p value** |
| --- | --- | --- | --- | --- | --- |
| **Site** | Factorial | Agricultural area (Intercept) | -4.119 | 0.751 | **<0.001* (a)** |
|  |  | Forest edge | -1.296 | 0.343 | **<0.001* (b)** |
| **Time** | Continuous | N/A | 0.812 | 0.219 | **<0.001* (b)** |
| **Time^2** | Continuous | N/A | -0.074 | 0.018 | **<0.001*** |
| **Site*Time** | Factorial: Continuous | Forest edge: Time | -0.219 | 0.143 | **0.112** |

Table S5. Summary of modelled coefficients for the nightly abundance of *An. balabacensis*

collected in each trap (HLC – Human Landing Catch, MBT – Monkey Baited Trap, HEN –

Human-baited Electrocuting Net, MEN – Monkey-baited Electrocuting Net, a – statistical

difference relative to zero, b – statistical difference relative to reference category, * – indicates

statistically significant difference)

| **Variable** | **Levels** | **Coefficient** | **SE** | **p value** |
| --- | --- | --- | --- | --- |
| **Trap** | HLC (reference) | -0.331 | -5.9492 | **0.001* (a)** |
|  | MBT | -1.611 | 1.1408 | **0.001* (b)** |
|  | HEN | N/A | N/A | **N/A** |
|  | MEN | N/A | N/A | **N/A** |

Table S6. Summary of modelled coefficients for the nightly abundance of *An. flavirostris* collected in each trap (HLC – Human Landing Catch, MBT – Monkey Baited Trap, HEN – Human-baited Electrocuting Net, MEN – Monkey-baited Electrocuting Net, a – statistical difference relative to zero, b – statistical difference relative to reference category, * – indicates statistically significant difference)

| **Variable** | **Levels** | **Coefficient** | **SE** | **p value** |
| --- | --- | --- | --- | --- |
| **Trap** | HEN (intercept) | -3.952 | 1.024 | **<0.001* (a)** |
|  | HLC | 3.145 | 1.021 | **<0.001* (b)** |
|  | MBT | 1.946 | 1.069 | **0.06 (b)** |
|  | MEN | -0.001 | 1.414 | **0.99 (b)** |

Table S7. Summary of modelled coefficients for the nightly abundance of *An. dispar* collected

in each trap (HLC – Human Landing Catch, MBT – Monkey Baited Trap, HEN – Human-baited

Electrocuting Net, MEN – Monkey-baited Electrocuting Net, a – statistical difference relative to

zero, b – statistical difference relative to reference category, * – indicates statistically significant

difference)

| **Variable** | **Type** | **Levels** | **Coefficient** | **SE** | **p value** |
| --- | --- | --- | --- | --- | --- |
| **Trap** | Factorial | HEN (intercept) | -2.482 | 0.656 | **<0.001* (a)** |
|  |  | MBT | 1.876 | 0.789 | **<0.001* (b)** |
|  |  | MEN | -0.212 | 0.865 | **0.80 (b)** |
|  |  | HLC | N/A | N/A | **N/A** |

Table S8. Summary of modelled coefficients for the nightly abundance of *An. greeni* collected in each trap (HLC – Human Landing Catch, MBT – Monkey Baited Trap, HEN – Human-baited Electrocuting Net, MEN – Monkey-baited Electrocuting Net, a – statistical difference relative to zero, b – statistical difference relative to reference category, * – indicates statistically significant difference)

| **Variable** | **Levels** | **Coefficient** | **SE** | **p value** |
| --- | --- | --- | --- | --- |
| **Trap** | HEN (intercept) | -3.545 | 0.755 | **<0.001* (a)** |
|  | MBT | 2.036 | 0.614 | **<0.001* (b)** |
|  | MEN | -0.405 | 0.912 | **0.65 (b)** |
|  | HLC | N/A | N/A | **N/A** |
