# Supplementary material for: The seasonal dynamics and biting behavior of potential Anopheles vectors of Plasmodium knowlesi in Palawan, Philippines
Source: Parasit Vectors. 2021 Jul 7;14:357. doi: 10.1186/s13071-021-04853-9 (PMC8261946; doi:10.1186/s13071-021-04853-9)
Supplement: Supplementary file 5 — Additional file 5: Table S1. Summary of total number of mosquitoes caught using the different trapping techniques in the trap comparison study. Table S2. Total number of female Anopheles mosquitoes collected in each trap and associated diversity indices (HLC – human landing catch, MBT – monkey-baited trap, HEN – human-baited electrocuting net, MEN – monkey-baited electrocuting net). [file 13071_2021_4853_MOESM5_ESM.docx]

**Additional File 5**

Table S1. Summary of total number of mosquitoes caught using the different trapping

techniques in the trap comparison study

| **Species** | **Trapping Techniques** | | | | **Total** | **% Composition** |
| --- | --- | --- | --- | --- | --- | --- |
|  | **HLC** | **MBT** | **HEN** | **MEN** |  |  |
| *An. balabacensis* | 30 | 6 | 0 | 0 | 36 | 0.55 |
| *An. flavirostris* | 23 | 7 | 1 | 1 | 32 | 0.49 |
| *An. dispar* | 0 | 60 | 13 | 7 | 80 | 1.21 |
| *An. greeni* | 0 | 23 | 3 | 2 | 28 | 0.42 |
| *An. franciscoi* | 0 | 6 | 2 | 2 | 10 | 0.15 |
| *An. ludlowae* | 4 | 10 | 3 | 2 | 19 | 0.29 |
| *An. samarensis* | 0 | 2 | 1 | 0 | 3 | 0.05 |
| *An. subpictus* | 0 | 0 | 0 | 1 | 1 | 0.02 |
| *An. tessellatus* | 2 | 0 | 0 | 0 | 2 | 0.03 |
| *An. vagus* | 0 | 2 | 1 | 0 | 3 | 0.05 |
| *An. vanus* | 0 | 9 | 4 | 2 | 15 | 0.23 |
| *Ae. albopictus* | 155 | 99 | 23 | 18 | 295 | 4.48 |
| *Ae. flavipennis* | 7 | 19 | 1 | 1 | 28 | 0.42 |
| *Ae. poicilius* | 2 | 5 | 2 | 2 | 11 | 0.17 |
| *Ae. vigilax* | 138 | 47 | 1 | 2 | 188 | 2.85 |
| *Ar. malayi* | 7 | 20 | 6 | 16 | 49 | 0.74 |
| *Ar. subalbatus* | 787 | 1,110 | 435 | 545 | 2,877 | 43.65 |
| *Cx. fuscocephala* | 1 | 15 | 0 | 0 | 16 | 0.24 |
| *Cx. gelidus* | 3 | 0 | 0 | 1 | 4 | 0.06 |
| *Cx. quinquefasciatus* | 0 | 1 | 0 | 0 | 1 | 0.02 |
| *Cx. vishnui* | 204 | 38 | 1 | 1 | 244 | 3.70 |
| *Anopheles* sp. | 1 | 2 | 1 | 0 | 4 | 0.06 |
| *Aedes* sp. | 54 | 64 | 2 | 11 | 131 | 1.99 |
| *Armigeres* sp. | 0 | 0 | 3 | 5 | 8 | 0.12 |
| *Culex* sp. | 511 | 1,109 | 148 | 143 | 1,911 | 28.99 |
| *Downsiomyia* sp. | 16 | 6 | 5 | 1 | 28 | 0.42 |
| *Lutzia* sp. | 0 | 2 | 0 | 0 | 2 | 0.03 |
| *Toxorhynchites* sp. | 0 | 3 | 0 | 0 | 3 | 0.05 |
| *Tripteroides* sp. | 1 | 1 | 0 | 0 | 2 | 0.03 |
| *Uranotaenia* sp. | 0 | 17 | 7 | 8 | 32 | 0.49 |
| *An. flavirostris* ♂ | 0 | 6 | 0 | 0 | 6 | 0.09 |
| *An. dispar* ♂ | 0 | 8 | 1 | 1 | 10 | 0.15 |
| *An. ludlowae* ♂ | 0 | 6 | 0 | 0 | 6 | 0.09 |
| *Anopheles sp.* ♂ | 0 | 2 | 1 | 1 | 4 | 0.06 |
| *Ae. albopictus* ♂ | 13 | 35 | 7 | 9 | 64 | 0.97 |
| *Ae. vigilax* ♂ | 0 | 1 | 0 | 0 | 1 | 0.02 |
| *Ar. subalbatus* ♂ | 8 | 125 | 37 | 21 | 191 | 2.90 |
| *Cx. vishnui* ♂ | 0 | 1 | 0 | 0 | 1 | 0.02 |
| *Aedes* sp. ♂ | 0 | 4 | 4 | 0 | 8 | 0.12 |
| *Armigeres* sp. ♂ | 0 | 0 | 8 | 0 | 8 | 0.12 |
| *Culex* sp. ♂ | 5 | 187 | 11 | 12 | 215 | 3.26 |
| *Downsiomyia* sp. ♂ | 0 | 1 | 0 | 0 | 1 | 0.02 |
| *Uranotaenia* sp. ♂ | 0 | 3 | 9 | 1 | 13 | 0.20 |
| **Grand Total** | **1,972** | **3,062** | **741** | **816** | **6,591** | **100.00** |

Table S2. Total number of female *Anopheles* mosquitoes collected in each trap and associated diversity indices (HLC – Human Landing Catch, MBT – Monkey baited Trap, HEN – Human-baited Electrocuting Net, MEN – Monkey-baited Electrocuting Net)

| ***Anopheles* species** | **HLC** | **MBT** | **HEN** | **MEN** |
| --- | --- | --- | --- | --- |
| **Total** | 59 | 125 | 28 | 17 |
| **Species Richness** | 4 | 9 | 8 | 7 |
| **Gini-Simpsons Diversity** | 0.60 | 0.72 | 0.75 | 0.81 |
| **G-S 95%CI** | <0.001 | <0.001 | <0.001 | <0.001 |
